# Metagenomic analysis of gut microbiota and its correlation with thyroid hormones in papillary thyroid cancer before and after operation

**DOI:** 10.1371/journal.pone.0356770

**Published:** 2026-08-24

**Authors:** Wenyi Qin, Jiajun Huo, Xiongsheng Xiao, Siyi Li, Haiqing Luo, Yongkang Wu, Weijie Chen, Zhuoting Chen, Changwei Zheng, Muge Liu, Baibei Li, Guiping Zhou, Zhibin Huang, Xuemeng Li, Jinquan Li, Zhi Zhang, Jian Ye

**Affiliations:** 1 Department of Thyroid and Vascular Surgery, Affiliated Hospital of Guangdong Medical University, Zhanjiang, China; 2 Department of Hepatobiliary Surgery, Zhanjiang Key Laboratory of hepatobiliary related diseases, Affiliated Hospital of Guangdong Medical University, Zhanjiang, China; 3 Zhanjiang Key Laboratory of Human Microecology and Clinical Translation Research, Guangdong Key Laboratory of Age-Related Cardiac and Cerebral Diseases, Affiliated Hospital of Guangdong Medical University, Zhanjiang, China; 4 Zhanjiang Key Laboratory of metabolic disease research, Affiliated Hospital of Guangdong Medical University, Zhanjiang, China; 5 Geriatric Medicine Center, Affiliated Hospital of Guangdong Medical University, Zhanjiang, China; Airlangga University Faculty of Medicine: Universitas Airlangga Fakultas Kedokteran, INDONESIA

## Abstract

**Background:**

The changes of intestinal flora and thyroid hormone levels before and after operation for papillary thyroid cancer (PTC) and their relationship are not clear. It may interfere with the intestinal thyroid axis, change the systemic thyroid hormone regulation and exogenous treatment response. It is recognized that this microbial involvement is clinically related to long-term metabolic outcomes, and future strategies for microbial targeted adjuvant therapy are suggested. This study aims to provide direct evidence and a comprehensive understanding of the relationship between intestinal flora and thyroid hormone levels before and after surgical treatment of papillary thyroid cancer through metagenomic analysis.

**Methods:**

As a paired before and after observation study, 20 patients diagnosed with papillary thyroid cancer were included in the study. Fecal samples and thyroid hormone levels were collected within 3 days before operation and 72 hours after operation. First, the intestinal microbiota of these 20 patients was analyzed for metagenomic differences, such as α and β diversity analysis, PCoA, ANOSIM, Spearman correlation analysis, FDR correction, KEGG pathway enrichment and correlation network analysis. Subsequently, the changes of hormone levels were examined in combination with the collected intestinal microbiota.

**Results:**

There were significant differences in the diversity and composition of the gut microbiota in patients with papillary thyroid cancer before and after surgery. At the species level, the eight most significantly different groups identified were *Bacteroides* sp*., Clostridium* sp*., Bacteroides fragilis, Alistipes* sp*., Parabacteroides* sp*., Bacteroides thetaiotaomicron, Phocaeicola dorei*, and *Oscillibacter* sp*.* Notably, *Clostridium* sp. higher abundance in the pre-operative group, whereas *Bacteroides* sp.*, Bacteroides fragilis, Alistipes* sp.*, Parabacteroides* sp.*, Phocaeicola* sp., and *Bacteroides thetaiotaomicron* were more prevalent in the post-operative group. After operation, FT4 showed an upward trend, while TSH, PTH and HTG showed a downward trend. The primary differential pathways associated with these changes pertained to iron uptake and regulation, polysaccharide utilization and transport, as well as metabolism and stress response. The pre-operative group was predominantly involved in ribosome biosynthesis, along with amino acid synthesis for valine and leucine. In contrast, the post-operative group primarily engaged in lipoic acid metabolism, glycosaminoglycan degradation, and bacterial secretion systems.

**Conclusion:**

This study found that the composition, diversity and function of intestinal flora changed in patients with papillary thyroid cancer after operation. Specific microbial taxa may be associated with fluctuations in thyroid hormone levels. In the future, regulating intestinal flora can be used as an adjuvant therapy for hormone regulation in patients undergoing PTC surgery.

## Introduction

In recent decades, thyroid cancer (TC) [1] has become more prevalent, ranking as a major endocrine malignancy and significant global health concern [2]. Papillary thyroid carcinoma (PTC), the most prevalent form of thyroid cancer, demonstrates indolent biological behavior and an excellent prognosis; however, patients face a significant risk of disease recurrence [3]. Studies have shown that in China and Korea, the incidence rate of thyroid cancer in women is higher than in other regions of the world [4].

Thyroid hormone (TH) [5] is one of the most important indicators for assessing thyroid function and includes tetraiodothyronine (thyroxine, T4) and triiodothyronine (T3). In vitro evidence indicates that thyroid hormones possess tumor-promoting capabilities, and the combination of increased thyroid hormone levels with decreased TSH concentrations may contribute to cancer development [6]. Therefore, in thyroid cancer patients, blood levels of T3 and T4 increase while TSH levels decrease [7]. The mainstay of PTC treatment at present is surgical resection [8], after which there is a significant reduction in thyroid hormone levels compared to the previous period and a significant increase in TSH levels, which may also lead to complications such as hypothyroidism [9]. As a result, the patient’s thyroid hormone levels will be lower than normal for a period of time after the operation, and thyroid hormone replacement therapy and regular follow-ups will be required.

With further research, the relationship between thyroid function, metabolism, and microbiota has been progressively discovered, and the gut-thyroid axis [10] has been modelled. Gut microbiota may influence thyrotropin secretion through the hypothalamic-pituitary axis [11]; Thyroid hormones cannot be synthesized without iodine and iron [12]; selenium and zinc play an important role in the conversion of T4 to T3, and both of these micronutrients are dependent on synthesis or absorption from the gut [13]. When these micronutrients are out of balance, the homeostasis of thyroid hormone production is disrupted. Some studies have shown a causal relationship between dysbiosis of the microbiota and thyroid cancer and suggest that monitoring the abundance of gut microflora could be a new indicator of thyroid cancer [14]. The thyroid gland can achieve homeostasis through the action of microbiota on the immune system and the metabolism of micronutrients; when the microbiota is out of balance, it may induce thyroid diseases such as thyroid cancer.

Our team previously conducted a metabolomic analysis of feces from patients with papillary thyroid cancer before and after surgery. We found that the upregulated lipid metabolites in the postoperative group were mainly enriched in the steroid hormone biosynthesis pathway, while the downregulated metabolites were significantly enriched in the arachidonic acid metabolism pathway [15]. Therefore, we speculate that the microbiota of patients changed before and after surgery, leading to changes in metabolites. In recent years, macro-genomics has been widely used for diagnosis and prognostic characterization of various cancers. Metagenomics has been used in the diagnosis and prevention of many types of cancer, including breast cancer and liver cancer. Combined analyses based on genomics and metabolomics can also reveal differences in the abundance and metabolites of microbiota at different stages of thyroid cancer and the molecular mechanisms of papillary thyroid carcinoma, thus improving the diagnosis and treatment of PTC and the long-term survival rate of patients [3,16]. Based on this, we used the same batch of fecal samples for metagenomic analysis, in order to find the microbiota that changed significantly before and after surgery.

Through genomics and metabolomic analysis, this experiment aims to explore the possible relationship between preoperative and postoperative changes in microbiota, metabolites, and hormone levels in patients with papillary thyroid carcinoma. On the basis of existing studies, this experiment will establish the relationship among microorganisms, metabolites and phenotypes, so as to improve our understanding of the changes of human hormone levels and microbiota before and after thyroid cancer surgery. This experiment will also influence the level of thyroid hormone through the changes of microbiota, and supplement the evidence supporting the role of the gut thyroid axis in the pathogenesis and regression of thyroid cancer.

## Materials and methods

### 1. Volunteer selection

As an exploratory and retrospective study, the original intention of this research design is to explore the changes in gut microbiota before and after surgery for papillary thyroid carcinoma and the possible relationship between gut microbiota and thyroid hormones. The volunteers were selected from 20 patients admitted to the Affiliated Hospital of Guangdong Medical University from 01/01/2022–31/12/2024 who were first diagnosed. Inclusion criteria are as follows: 1) For patients diagnosed for the first time and diagnosed with primary papillary thyroid carcinoma based on pathological results without lymph node or distant metastasis, the subsequent treatment plan is to choose subtotal thyroidectomy for thyroid cancer; 2) Prior to admission, no treatment was received and patients with autoimmune thyroid disease were excluded; 3) Patient informed consent, voluntary participation and signing of informed consent form. Exclusion criteria were as follows: 1) Volunteers with cancers other than primary thyroid cancer; 2) Volunteers with a history of alcohol or narcotic abuse, drug abuse, or a history of psychiatric disorders (e.g., schizophrenia, obsessive-compulsive disorder, depression), oppositional personalities, poor motivation, suspiciousness, or other emotional or intellectual problems that might affect the informed validity of participation in this study; 3) Volunteers with other endocrine related diseases (S1 Fig). The volunteers ranged in age from 27 to 61 years old, and had completed pre-operative and post-operative baseline data, including age, gender and various physiological indexes, and the volunteers were followed up (S1 Table). This study was ethically reviewed by the Clinical Research Ethics Review Committee of the Affiliated Hospital of Guangdong Medical University, Ethical Approval No. PJ2021−079. All subjects signed an informed consent form.

### 2. Fecal samples collection

According to the sample collection instructions, volunteers collected fecal samples in the hospital. The pre-operative group (FF group) included fecal samples collected from 20 patients within 3 days before surgery, and the post-operative group (BF group) included fecal samples from the same 20 patients within 72 h after surgery. Fecal samples were collected using disposable fecal collection cups before antibiotic use and immediately after defecation, and stored in a refrigerator at 4 °C. Researchers transported fecal samples to the research center using a 0 °C freezer within 8 h. After completing the packaging, store the fecal samples in the 80 °C refrigerator at the Affiliated Hospital of Guangdong Medical University.

The collection of postoperative fecal samples within 72 h after surgery was due to significant changes in the composition of the gut microbiome during the first 72 h after major physiological stress, with microbial diversity reaching its lowest point around 72 h after surgery. Capturing the early stages of microbiome disruption is crucial for establishing baseline trajectories of postoperative microbiota dynamics and identifying potential early biomarkers that may provide information for timely treatment interventions

### 3. DNA extraction and library construction and quality control

Take 0.2 grams of fecal sample and use Magen’s HiPure Stool DNA Fecal Genome Extraction Kit (item number D3141) to extract total microbial DNA from the fecal samples. The kit comes with a dedicated inhibitor removal reagent, which can effectively remove PCR inhibitory impurities such as polysaccharides, bile salts, and mucus from feces, obtaining high-purity microbial total DNA. After the extraction is completed, DNA quantification and integrity grading quality control will be carried out uniformly. The grading standards are as follows: Grade A: total DNA content ≥ 200 ng, main fragment ≥ 500 bp, no/mild impurity RNA contamination, and can be directly used for routine library construction; 50 ng ≤ total DNA < 200 ng, only single library construction can be completed; Grade C: 20 ng ≤ total DNA < 100 ng, fragment ≥ 250 bp, there is a risk of library construction. 5 ng ≤ total DNA < 20 ng, fragment ≥ 500 bp, it is recommended to use transposase library construction; Grade D: Total DNA content<5 ng or fragment<250 bp, severe contamination, re sampling is required. Communicate separately to confirm the feasibility of library construction for detecting abnormal or special matrix samples. The qualified DNA was constructed using the Novozymes VAHTS Universal Plus DNA Library Prep Kit for Illumina V2 (ND627), which integrated DNA fragmentation, end repair, A-tail and adapter ligation. The Illumina sequencing library was obtained by PCR enrichment and magnetic bead purification. Use ultrasound treatment or enzyme digestion to fragment DNA to a length suitable for sequencing. The DNA fragment is repaired at the end to ensure that the end is flat. By using high fidelity DNA polymerase for PCR amplification, connecting sequencing adapters and enriching DNA fragments with adapters; Use magnetic beads or columns to purify and remove non luminescent adapters and primers. Measure the concentration of the library using quantitative fluorescence PCR (qPCR) and check the size distribution of library fragments using a biological analyzer or fragment analyzer to confirm successful library construction.

### 4. Raw data quality assessment and filtering

FastQC (Version 0.12.1) was used to evaluate the quality of the raw sequencing data (reads), and the sequencing quality of all samples met the universal quality standards. Subsequently, Fastp (Version 0.23.2) was used to remove potential human sequence contamination from the sample, and Bowtie2 (Version 2.3.5.1) was used to align the reads to the human reference genome GRch38, while retaining the reads that were not aligned for further analysis.

### 5. Sequencing on board

Dilute the library to the appropriate concentration and load it into the sequencing chip or flow-through tank. Use the sequencer NovaSeq 6000 with the sequencing mode set to PE150 to start the sequencing reaction, and the device records the base signals in real time and generates raw data.

### 6. Bioinformatics and statistical analysis

Quantitative demographic and clinical characteristics with nonnormal distributions are expressed as the medians (P25, P75), and differences between groups were tested using the Wilcoxon rank sum test. Results with p < 0.05 were considered statistically significant.

For metagenomic sequencing data, raw paired-end reads were first quality‑filtered using Trimmomatic (v0.39) to remove adapters and low‑quality bases. Clean reads were then assembled into contigs using MEGAHIT (v1.2.9) with default parameters. Open reading frames were predicted on contigs ≥ 500 bp using Prodigal (v2.6.3), and predicted genes were translated into protein sequences. Redundant genes were clustered at 95% identity using CD‑HIT (v4.8.1) to construct a non‑redundant gene set. For taxonomic profiling, high‑quality reads were aligned against the non‑redundant gene set using Bowtie2 (v2.3.5.1), and the abundance of each gene was calculated as transcripts per million (TPM). Species‑level classification was performed by mapping the gene set to the NCBI NR database using DIAMOND (v2.0.15) with an e‑value threshold of 1e‑5; the Lowest Common Ancestor (LCA) algorithm in MEGAN (v6.25.7) was then applied to assign each gene to its most specific taxonomic node. Relative abundance of each species was derived by summing the TPM values of genes assigned to that species. Alpha diversity analysis was calculated using Mothur software (version 1.30.2), and intergroup differences were compared using the Wilcoxon rank‑sum test. Microorganism community structure analysis was performed using R software (version 3.3.1). Principal coordinate analysis (PCoA) based on Bray–Curtis distance was conducted to examine the similarity of microorganism community structures among samples. Combined with ANOSIM tests, the significance of differences in community structure between groups was evaluated. Community composition bar charts were plotted at the family, genus, and species levels, respectively. Species with an abundance ranking below 30 are classified as “Others”. Species‑level diversity analysis was conducted based on species classification and abundance data derived from non‑redundant genes. The Wilcoxon rank‑sum test was performed using the stats package in R and the scipy package (v1.0.0) in Python. Further analyses were conducted using the R package vegan (version 2.4.3) to perform Mantel tests (network heatmaps) and redundancy analysis (RDA). Spearman correlation analysis was employed to evaluate associations between species and clinical indicators, with p‑values corrected using the FDR method. KEGG pathway enrichment analysis was performed using the ReporterScore package (version 3.3.1) in R, where functional annotations of the non‑redundant gene set were first obtained by searching against the KEGG GENES database (release 110.0) using DIAMOND, and pathway abundances were subsequently calculated. Correlation network analysis screened species based on Spearman’s correlation coefficient (|r| > 0.5 and p < 0.05). Network diagrams were constructed using Python’s Networkx package (version 1.11), and species and functional contribution analyses were performed using Python (version 2.7.0). The p‑value was used for differential abundance analysis and KEGG pathway analysis in other microbial communities.

## Results

### 1. Changes in gut microbiota in patients with PTC before and after surgery

To determine whether the gut microbiota composition of PTC patients changes before and after surgery, we examined the fecal microbiology of 20 patients before and after surgery.

**Fig 1:**
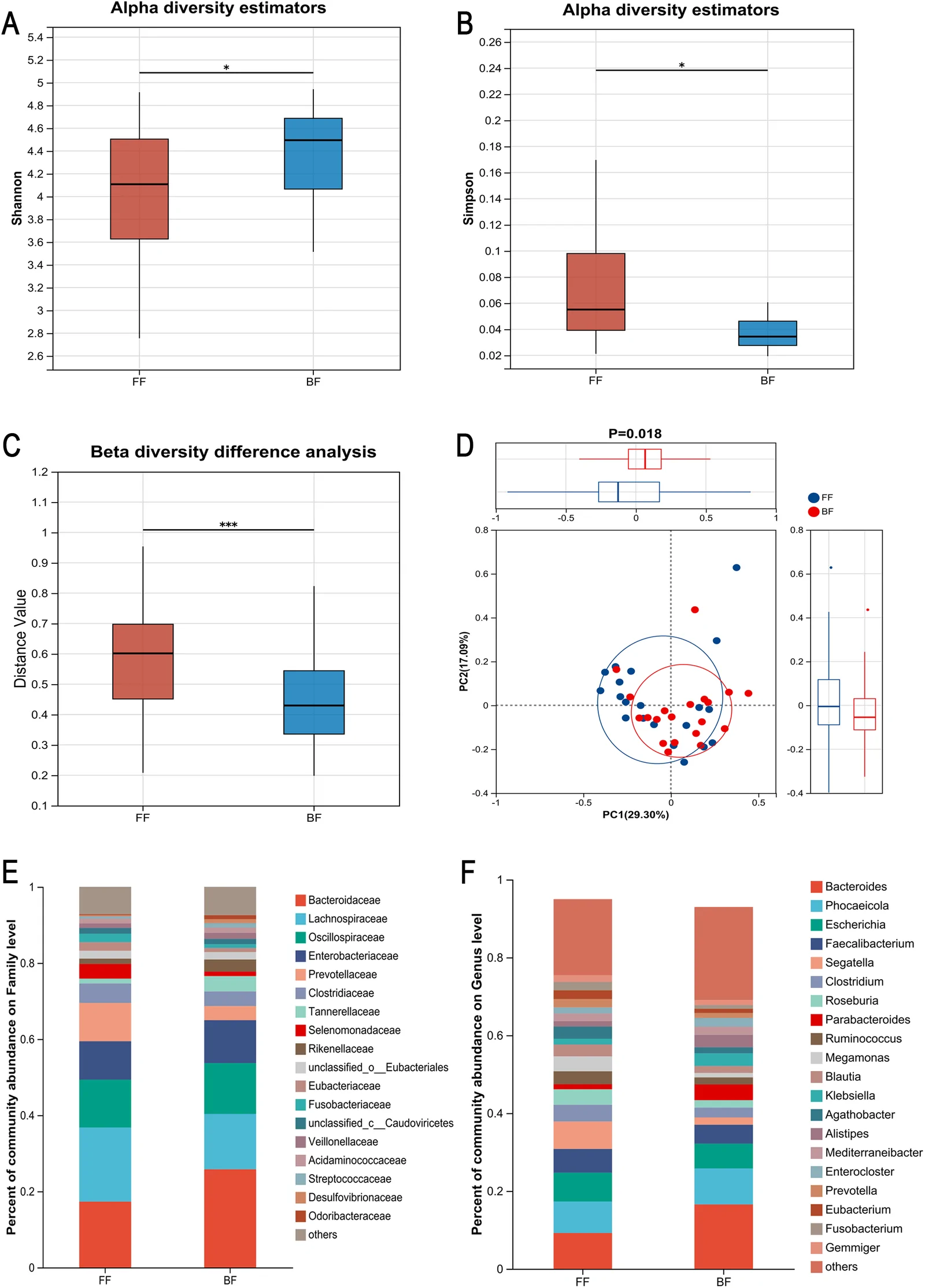
Changes in the gut microbiota: (A) α-Diversity based on the Shannon index between the groups; (B) α-diversity based on the Simpson index between the groups; (C) β-diversity based on the Bray–Curtis distance at the family level; (D) PCoA scatter plot at the family level; Microbiota composition at the family level; (F) Microbiota composition at the genus level. FF stands for pre-operative group, and BF stands for post-operative group. Differences between groups were tested using the Wilcoxon rank sum test. *p < 0.05, **p < 0.01, ***p < 0.001. PCoA: Principal Coordinate Analysis.

The Alpha diversity results revealed that the Shannon index was significantly higher in the post-operative group (BF group) compared to the pre-operative group (FF group). Conversely, the Simpson index was significantly higher in the post-operative group than in the pre-operative group, indicating increased gut microbiota diversity in patients following surgery (Fig 1A and 1B). At the family level, analysis of intergroup differences in Beta diversity revealed significant disparities in the distribution of gut microbiota structures between the two groups (Fig 1C). Concurrently, PCoA analysis also demonstrated differences in the composition of microbiota communities between the two groups (Fig 1D). In the post-operative group, the abundance of *Bacteroidaceae, Tannerellaceae, Odoribacteraceae*, and other genera was higher than in the pre-operative group, while the abundance of *Prevotellaceae, Selenomonadaceae, Rikenellaceae*, and other genera was lower than in the pre-operative group (Fig 1E). At the genus level, the post-operative group exhibited higher abundance proportions of *Bacteroides, Parabacteroides, Klebsiella,* and *Alistipes* compared to the pre-operative group, while *Segatella, Megamonas, Fusobacterium,* and *Eubacterium* showed lower abundance proportions than the pre-operative group. *Bacteroides, Phocaeicola,* and *Escherichia* accounted for significant proportions in both groups, reflecting the critical role of these three genera in maintaining gut microbiota homeostasis (Fig 1F).

### 2. Genome sequencing reveals differences in gut microbiota composition before and after surgery in patients with PTC

**Fig 2:**
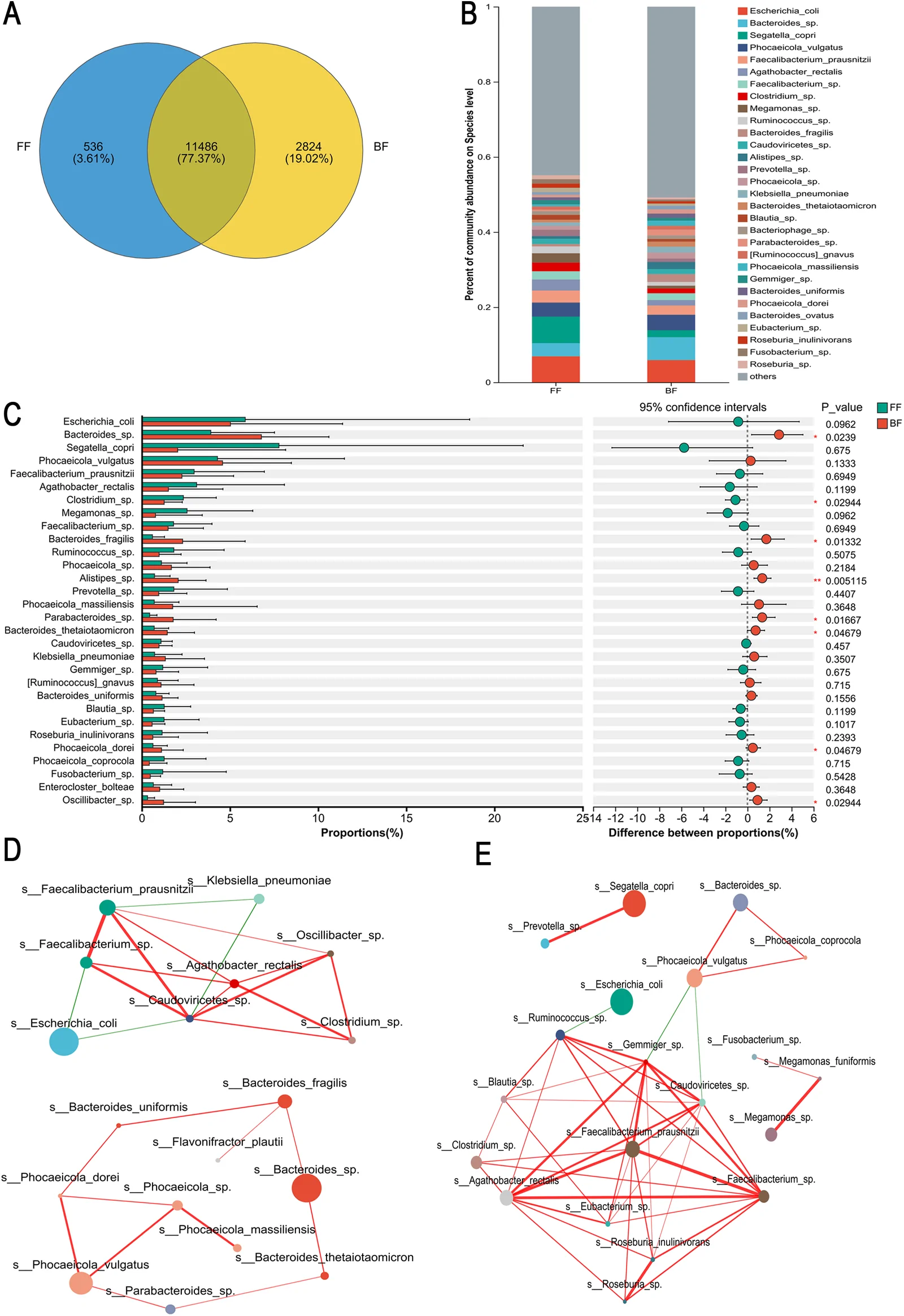
Microbiota differences based on metagenomic sequencing at the species level: (A) Venn diagram; (B) Microorganism composition; (C) Comparison of differences in the Top 30 abundance-ranked gut microbiota between the FF and BF groups; (D, E) Network diagram of the Top 20 microbiota communities by abundance in the BF group (D) and FF group (E). In the network diagram, larger nodes indicate higher abundance values for the corresponding species. Species belonging to the same phylum are represented by nodes of the same color. Red lines denote positive correlations between species, while green lines indicate negative correlations. Thicker lines signify stronger correlations between species, and a greater number of lines indicates more extensive connections between a species and others. Differences between groups were tested using the Wilcoxon rank sum test and only results with correlation coefficients >0.5 and *p*-values < 0.05 are shown. FF stands for pre-operative group, and BF stands for post-operative group.

To determine changes in the gut microbiome at the species level in patients with PTC before and after surgery, metagenomic sequencing was performed on fecal sampless from 20 pairs of patients with papillary thyroid carcinoma. Analysis of intergroup differences in Beta diversity, PCoA analysis, and ANOSIM analysis revealed differences in the distribution of microorganism community structures between the two groups at the species level (S2A–S2C Fig). The Venn diagram shows that the two groups share 11,486 species, with the pre-operative group containing 536 unique species and the post-operative group containing 2,824 unique species (Fig 2A). As shown in the community bar chart (Fig 2B) and single-group pie charts (S2D–S2E Fig), the abundance proportions of *Bacteroides* sp.*, Bacteroides fragilis, Alistipes* sp.*, Parabacteroides* sp.*, Phocaeicola* sp.*,* and *Bacteroides thetaiotaomicron* were significantly higher than in the pre-operative group, while the abundance of *Segatella copri, Clostridium* sp.*, Agathobacter rectalis, Megamonas* sp.*, Blautia* sp.*,* and *Phocaeicola dorei* was significantly lower than in the pre-operative group. Further differential analysis of the top 30 most abundant microbiota species revealed significant differences between the two groups in *Bacteroides* sp.*, Clostridium* sp.*, Bacteroides fragilis, Alistipes* sp.*, Parabacteroides* sp.*, Bacteroides thetaiotaomicron, Phocaeicola* dorei*,* and *Oscillibacter* sp. (Fig 2C). The univariate network diagram shows that in the pre-operative group, *Bacteroides* sp. serves as the core microbiota and exhibits direct or indirect positive correlations with *Bacteroides fragilis, Phocaeicola* dorei*,* and *Parabacteroides* sp. (Fig 2D); whereas in the post-operative group, *Segatella* copri constitutes the core microbiota (Fig 2E).

This indicates that the core gut microbiota of patients with papillary thyroid carcinoma undergoes changes following surgical treatment. Therefore, we hypothesize that these microorganism communities play a role in the alterations of thyroid hormones observed in these patients before and after surgery.

### 3. Correlation analysis between microbiota and thyroid hormone levels

We examined the changes in thyroid hormones and related biomarkers in 20 patients with papillary thyroid carcinoma before and after surgery. Specifically, we assessed the levels of free triiodothyronine (FT3), free thyroxine (FT4), thyroid-stimulating hormone (TSH), anti-thyroglobulin antibodies (A-TG), parathyroid hormone (PTH), and human thyroglobulin (HTG). All patients were prescribed levothyroxine sodium post-operatively in accordance with clinical guidelines. Statistical analysis revealed distinct trends in these biomarkers following surgery. The levels of FT4, FT3 and A-TG exhibited an increasing trend, while TSH, PTH, and HTG showed a decreasing trend Among them, FT4, TSH, PTH, and HTG showed significant differences before and after surgery, but FT3 and A-TG did not (Table 1).

**Table 1:** Baseline thyroid hormone characteristics of patients.

Hormone	FF (n = 20)	BF (n = 20)	*p*-value
TSH (mIU/L)	1.43 (0.98, 1.90)	1.35 (0.14, 1.76)	0.01324*
FT4 (pmol/L)	17.27 (16.05, 18.45)	21.53 (20.80, 23.15)	0.00001***
PTH (pg/ml)	48.07 (30.1, 49.0)	27.84 (20.65, 33.5)	0.00037***
HTG (ng/ml)	44.87 (10.8, 56.55)	4.76 (1.7, 6.09)	0.00002***
FT3 (pmol/L)	4.84 (4.39, 5.22)	5.07 (4.57, 5.53)	0.13049 ^ns^
A-TG (mmol/L)	25.65 (1.38, 23.68)	56.16 (15.00, 22.10)	0.09692 ^ns^

FF stands for pre-operative, and BF stands for post-operative. The hormone level is represented by M (Q_1_, Q_3_).

**Fig 3:**
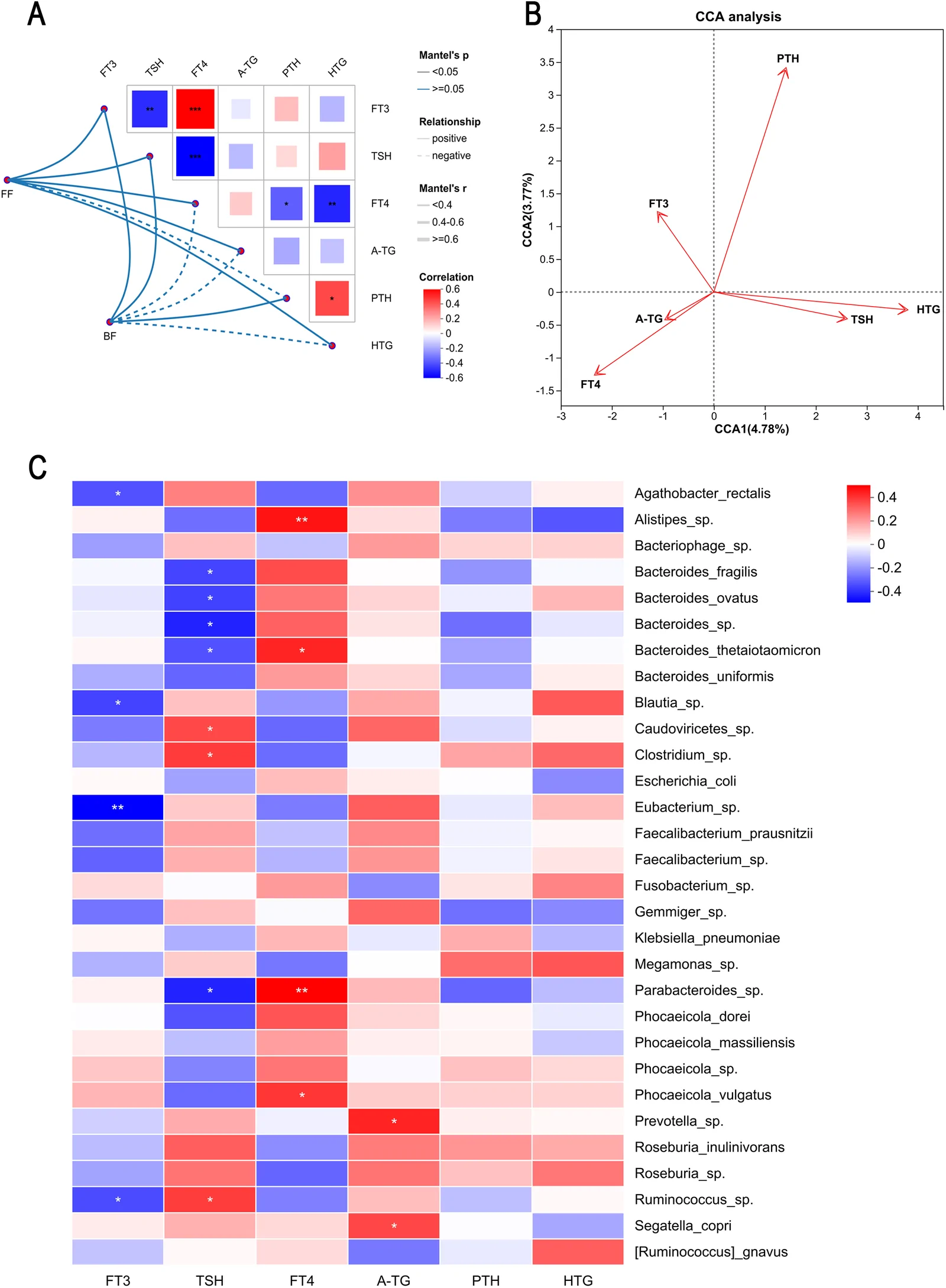
Analysis of the correlation between microbiota at the species level and hormone levels: (A) Mantel-test network heatmap of microbiota and thyroid hormones; (B) RDA/CCA plot of thyroid hormones, the angle between hormones represents their correlation, with acute angles indicating positive correlation and obtuse angles indicating negative correlation; (C) Heatmap plot of correlation between microbiota and thyroid hormones. Differences between groups were tested using the Wilcoxon rank sum test. *p < 0.05, **p < 0.01, ***p < 0.001, and p-values adjusted using the FDR method. RDA: Redundancy Analysis; CCA: Canonical Correspondence Analysis; ANOSIM: Analysis of Similarities; FDR: False Discovery Rate.

The Mantel-test network heatmap revealed negative correlations between microbiota and FT3 and PTH, as well as positive correlations between microbiota and A-TG and PTH before and after surgery. On the contrary, TSH and FT4 were positively correlated pre-operatively and negatively correlated postoperatively. The combined RDA/CCA plot shows a significant negative correlation of TSH with FT3 and FT4, as well as a significant positive correlation of both FT3 and FT4 (Fig 3A and 3B). We generated a correlation heatmap by comparing microbiota with significant differences to distinct thyroid hormone indicators. *Bacteroides* sp. and *Parabacteroides* sp. showed negative correlations with TSH; *Bacteroides thetaiotaomicron* and *Parabacteroides* sp. showed positive correlations with FT4 and *Eubacterium* sp. showed negative correlations with FT3 (Fig 3C). Based on these results, we speculate that there is a certain relationship between the microbiota and thyroid function, and changes in the gut microbiota may affect the secretion of thyroid hormones to some extent.

### 4. Functional changes in the gut microbiota of patients with papillary thyroid cancer before and after surgery

**Fig 4:**
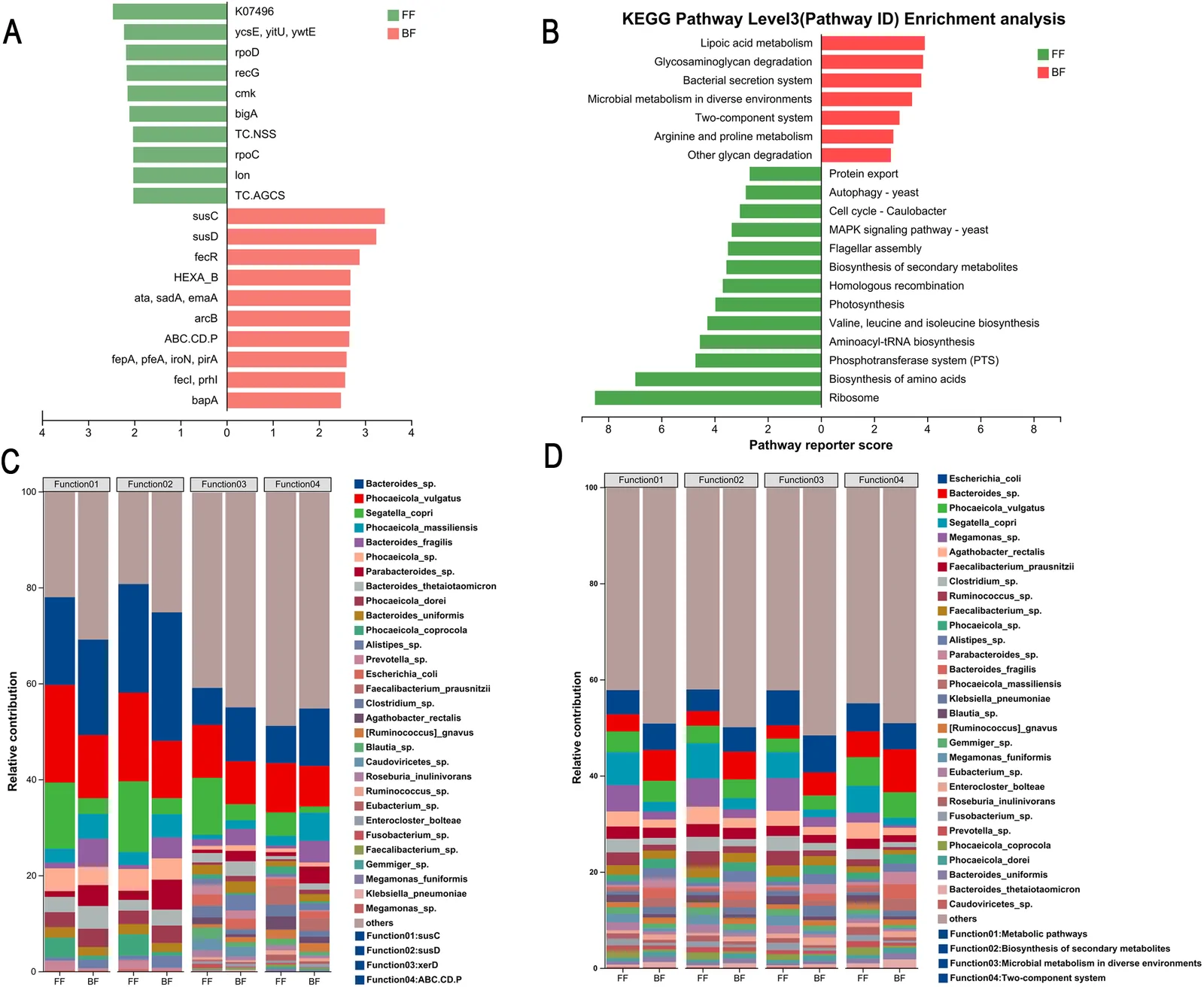
KEGG functional annotation of microbiota at the species level: (A) LEfSe discriminant bar chart of KEGG Name, the horizontal axis represents LDA scores and higher LDA scores indicate a greater influence of species abundance on the effect of variation; (B) KEGG Pathway Level3 (Pathway ID) Enrichment Analysis bar chart; (C, D) Bar chart showing functional contributions of the top 30 most abundant gut microbes at KEGG Name (C) and KEGG Level3 (D). FF stands for pre-operative group, and BF stands for post-operative group. LEfSe: Linear Discriminant Analysis Effect Size; LDA: Linear Discriminant Analysis; KEGG: Kyoto Encyclopedia of Genes and Genomes.

This study further annotated the microbiota metagenomic data using the KEGG database to determine the functional characteristics of microbiota in patients with papillary thyroid carcinoma before and after surgery. LEfSe analysis was used to process the samples and identify features with significant differences between the groups. The results indicate that the gut microbiota in the pre-operative group showed a bias toward environmental stress tolerance and basal metabolism. In contrast the post-operative group exhibited a bias toward polysaccharide and protein degradation capabilities, iron competition, and adhesion and colonization abilities (Fig 4A). Enrichment analysis of KEGG Pathway Level 3 (Pathway ID) revealed that significant pathways in the pre-operative group primarily involved ribosome and amino acid biosynthesis (e.g., valine and leucine). Gut microbiota functions may have shifted toward degrading the host mucosal layer to acquire nutrient and activate pathogenic pathways, such as secretion systems. Significant pathways in the post-operative group primarily involved fatty acid metabolism, glycosaminoglycan degradation, and bacterial secretion systems. Gut microbiota functions may have become more focused on basal metabolism and growth, relying on external nutrients such as diet (Fig 4B). The species contribution analysis diagram indicates that *Bacteroides* sp.*, Bacteroides fragilis, Alistipes* sp.*, Parabacteroides* sp., and *Bacteroides thetaiotaomicron* contributed more to the post-operative group than the pre-operative group. In contrast, *Clostridium* sp. showed the opposite pattern (Fig 4C and 4D). These results reveal a fundamental shift in gut microbiota function before and after surgery and demonstrate that changes in gut microbiota abundance alter their functional modes, thereby influencing thyroid hormone secretion.

## Discussion

Growing research has focused on the possible regulatory effects of microbiota communities, and their metabolic byproducts on thyroid hormone homeostasis in PTC patients during the perioperative period [17–19]. Current data indicate that dysbiosis of the intestinal microbiota may exert substantial effects on thyroid hormone regulation. Comparative analyses have revealed characteristic gut microbiome profiles in thyroid cancer patients that differ markedly from healthy individuals, suggesting a potential involvement of gut microbes in thyroid carcinogenesis [14]. Similar findings have also been observed in saliva microbiota, further supporting the gut-thyroid axis. However, the specific mechanisms by which gut microbiota influence thyroid hormone changes in PTC patients before and after surgery remain poorly understood, and research into what role the microbiota play in the changes in thyroid hormones associated with pre- operative and post- operative papillary thyroid cancer is still limited. Therefore, in-depth classification and functional characterization of the gut microbiota in PTC patients before and after surgery are essential. This study aims to fill this gap by analyzing the metagenomic data of gut microbiota in 20 PTC patients. The results reveal significant effects of surgery on the composition, diversity, and functionality of the gut microbiota, and provide insights into the potential associations between gut microbiota and thyroid hormone levels.

### 1. Impact of surgery on the diversity and composition of microbiota

The richness and diversity of gut microbiota are crucial for maintaining the metabolic balance of the body’s hormones [20]. Our analysis revealed a significant elevation in microbiota alpha diversity following surgical intervention, demonstrated by increased Shannon indices and reduced Simpson indices. These metrics collectively indicate enhanced species richness and more even community distribution post-operatively. The observed microbial changes may be caused by the intestinal microenvironment changes caused by surgery. Therefore, we speculate that there may be some relationship between the changes of microbiota and the changes of hormones after surgery, but this hypothesis still needs more experiments to verify. These findings corroborate existing literature documenting how surgical procedures can influence gut microbiota composition through immunomodulatory effects or alterations in host metabolism [21]. In addition, β diversity analysis showed significant differences in the structure of gut microbiota before and after surgery, further emphasizing the partial impact of surgery on gut microbiota. However, the effects of perioperative fasting, surgical stress, anesthesia, and exposure to levothyroxine on gut microbiota cannot be ignored. Our samples were all from the same treatment group in the same center, and their management during the perioperative period was basically the same, minimizing the impact of the above factors on the experiment These results collectively support the existence of an association between changes in hormone levels and gut microbiota in patients with papillary thyroid cancer before and after surgery. Regarding the composition of the microbiota, we found that the abundance of *Bacteroidaceae* and *Tannerellaceae* increased in the post-operative group, while the abundance of *Prevotellaceae* and *Selenomonadaceae* decreased. This shift may be related to the initiation of thyroid hormone replacement therapy after surgery, such as levothyroxine sodium [22]. Previous studies have shown that changes in thyroid hormone levels can influence the metabolic environment of gut microbiota [23]. At the genus level, the abundance of *Bacteroides* and *Parabacteroides increased significantly, while the abundance of Megamonas* and *Eubacterium* decreased. These findings suggest that these genera may play a pivotal role in the reconstruction of gut microecology during the post-operative period and may also contribute to changes in thyroid hormone levels.

### 2. Correlation between microbiota and thyroid hormones

Through correlation analysis, we found significant associations between *Bacteroides fragilis*, *Bacteroides thetaiotaomicron, Parabacteroides* sp. and *Clostridium* sp. and thyroid hormone levels [24]. For example, *Parabacteroides* sp. showed a positive correlation with FT4 and a negative correlation with TSH; *Bacteroides thetaiotaomicron* also exhibited a positive correlation with FT4. These findings align with the physiological manifestations of hyperthyroidism, characterized by elevated FT4 and reduced TSH levels [25]. This suggests that these bacterial genera may influence postoperative thyroid function recovery by regulating host thyroid hormone metabolism, indicating that gut microbiota may affect the expression of thyroid-related proteins through immune modulation or direct metabolic pathways [26]. This discovery opens up a new perspective for the mechanism study of the relationship between microbiota and postoperative recurrence/prognosis of thyroid cancer. At the same time, it should be clarified that the idea of using gut microbiota to assist rehabilitation, delay recurrence, or shorten treatment cycles is still within the scope of scientific hypotheses and has not yet reached the stage of clinical translation.

### 3. Functional changes in microbiota

As an important part of the microbiota in the human body, the microbiota has received widespread attention for its impact on human health. KEGG functional annotation analyses showed significant changes in metabolic pathways in the microbiota of post-operative patients. The increased abundance of pathways associated with iron uptake, polysaccharide utilization and stress response [27] in the post-operative group may be related to the increased nutritional requirements of the body or the modulation of inflammatory responses after surgery. For example, enrichment of iron uptake pathways may reflect adaptive adjustments in iron metabolism by the microbiota in the post-operative anemic state [28]. Furthermore, the high contribution of *Bacteroides* sp.*, Bacteroides fragilis, Parabacteroides* sp.*, and Bacteroides thetaiotaomicron* to polysaccharide metabolism pathways such as susC and susD further supports the crucial role of these bacterial genera in post-surgical intestinal energy metabolism. The negative correlation of Eubacterium with certain metabolic pathways may be related to its predominance in the pre-operative gut, being weakened by post-operative environmental changes.

Meanwhile, analysis of the post-operative group revealed that alpha-lipoic acid metabolism and arginine/proline metabolism play crucial roles in the recovery process. Integrating these findings with our group’s prior KEGG enrichment analysis of metabolites in patients with papillary thyroid carcinoma before and after surgery: Upregulated lipid metabolites in the post-operative group were primarily enriched in Steroid hormone biosynthesis pathways. Downregulated metabolites showed significant enrichment in Arachidonic acid metabolism pathways [15]. This indicates close interactions between different metabolic pathways.

The synthesis of steroid hormones is a highly energy-consuming process that relies on substantial ATP to drive it. Alpha-lipoic acid, as an essential cofactor for both the pyruvate dehydrogenase complex and the α-ketoglutarate dehydrogenase complex, directly influences the entry point and critical steps of the tricarboxylic acid cycle, thereby regulating ATP production efficiency. Simultaneously, alpha-lipoic acid exerts a protective effect on cells synthesizing steroid hormones, ensuring sustained cellular function [29]. The NO produced by arginine metabolism inhibits the activity of steroid synthase, while steroid hormones rely on collagen rich in proline to promote growth, indirectly increasing the demand for proline utilization.

On the other hand, arachidonic acid [30] generates large amounts of reactive oxygen species during the metabolic processes of cyclooxygenase and lipoxygenase, leading to cellular oxidative stress. Alpha lipoic acid alleviates oxidative damage in arachidonic acid metabolism through its antioxidant effect, while NO derived from arginine can regulate the metabolic effect of this metabolite through the generation of reactive oxygen species signaling molecules, and directly bind to COX enzymes to affect prostaglandin synthesis. During tissue repair, inflammation and repair signals driven by the arachidonic acid pathway promote collagen and extracellular matrix synthesis, thereby increasing the demand for proline [31].

In summary, we believe that changes in postoperative metabolite and amino acid levels are closely associated with the patient’s recovery process. These alterations may support postoperative tissue repair and functional recovery by reducing inflammatory responses and promoting the synthesis of collagen and extracellular matrix.

### 4. The correlation between surgery, thyroid hormones, gut microbiota and their functions

Thyroidectomy has been widely confirmed to cause significant changes in thyroid hormone levels in patients [32]. Our latest research has found significant changes in the microbial community structure and diversity of patients after radical thyroidectomy. The early postoperative changes in gut microbiota may be attributed more to multiple factors during the perioperative period, such as fasting, surgical stress, anesthesia, antibiotic use, hospital diet, abnormal intestinal motility, inflammatory response, and exposure to levothyroxine, rather than the unique biological effects of thyroidectomy itself. However, this observation prompts us to hypothesize whether surgical intervention may affect the metabolism of thyroid hormones by reshaping the composition of the gut microbiota? Based on the theoretical framework of the gut-thyroid axis [10,33], gut microbiota influence nutrient absorption and hormone synthesis, as well as the efficiency of trace element uptake, including iodine, selenium, and zinc. As a vital immune organ, the gut modulates immune system function through its microbiota, helping to prevent excessive immune activation. Furthermore, short-chain fatty acids produced by gut microbiota metabolism enhance intestinal barrier function, reduce systemic inflammation, and indirectly maintain thyroid health via immune regulatory mechanisms [34]. Combined with our observed correlation between gut microbiota and thyroid hormone levels, we propose an innovative hypothesis. The gut microbiota community may play a key role in the regulating of thyroid function after surgery. Specifically, microbiota disorder caused by surgery may affect thyroid hormone levels through the following pathways, such as altering the absorption of trace elements like iodine, selenium, and zinc [35] or by influencing the enterohepatic circulation of thyroid hormones [18].

Studies have shown that there are significant differences in intestinal gut composition between patients with thyroid cancer and healthy people. Specifically, in the disease group, the relative abundance of *Bacteroides* and *Parabacteroides* decreased significantly, while the abundance of *Eubacterium* increased significantly. It is worth noting that this study found that patients with thyroid cancer after surgery showed a similar trend of flora changes: the content of *Bacteroides* and *Parabacteroides* decreased, and the content of *Eubacterium* increased. This discovery is consistent with previous research. It may suggest a correlation between the occurrence of cancer and structural changes in specific microbial communities; The phenomenon of postoperative microbiota shifting towards a healthy direction may reflect a compensatory adjustment trend in the internal environment of the body after surgical resection of the lesion. These results may provide indirect support from a microbiome perspective for the effectiveness of surgical resection in cancer treatment. In addition, we speculate that surgical intervention may help promote a shift in patients’ gut microbiome characteristics towards a healthier state.

*Bacteroides* is one of the most abundant and core genera in the gut of healthy adults, capable of producing short-chain fatty acids, such as acetate and propionate. These substances exert anti-inflammatory effects and help maintain the intestinal barrier [36]. However, during dysbiosis and increased intestinal permeability, excessive lipopolysaccharides entering the circulatory system trigger systemic, chronic, low-grade inflammation. This, in turn, promotes the development of thyroiditis and even thyroid cancer [37,38]. Patients often develop hypothyroidism postoperatively, leading to routine clinical supplementation with levothyroxine for treatment. Hypothyroidism causes decreased gastrointestinal motility, potentially providing more substrates for *Parabacteroides*, which primarily relies on carbohydrates as its nutrient source, and promoting its overgrowth. *Parabacteroides* overgrowth is associated with low-grade inflammation and insulin resistance, and may interfere with the intestinal absorption of levothyroxine, thereby affecting its therapeutic efficacy [39,40]. However, since postoperative sampling occurs within 72 hours, this mechanism tends to be viewed as a hypothesis for long-term risk or future longitudinal tracking. *Eubacterium* is one of the key beneficial bacteria that produces butyrate, which can be considered a supporter of thyroid health. Butyrate not only directly energizes colon cells, strengthening and repairing the intestinal barrier, but also possesses potent anti-inflammatory effects, regulating T-cell function and promoting immune tolerance [41]. Adequate levels of *Eubacterium* and its metabolite butyrate help mitigate inflammatory immune attacks on the thyroid while maintaining a healthy gut environment, thereby ensuring efficient absorption of nutrients and medication [42–44].

Although existing studies have demonstrated a relationship between gut microbiota and thyroid diseases [45], literature directly verifying this mechanism is currently limited. Based on existing evidence, we speculate that the gut microbiota may play a role in hormone regulation during the perioperative period of thyroid cancer. Further elucidating this regulatory network may help to understand the potential molecular mechanisms underlying postoperative thyroid function compensation. We hypothesize that in the future, targeted regulation of gut microbiota may provide ideas for optimizing postoperative hormone replacement regimens, theoretically promoting patients to reach physiological compensation more quickly.

### 5. Research limitations and Future research directions

The study systematically revealed changes in microbiota and their correlation with thyroid function before and after surgery in patients with papillary thyroid cancer, providing a new perspective for understanding the microbiological mechanisms that regulate thyroid function after surgery. However, this study still has several limitations.

Firstly, the sample size is extremely limited (only 20 cases), which is the most prominent shortcoming of this exploratory study. Such a small sample size not only reduces the effectiveness of statistical testing and increases the risk of false positive or false negative results, but also results in significant sampling errors in estimating any inter group differences or association strength, making it difficult to ensure the reproducibility of results in different populations. Therefore, the universality of the current findings should be treated with caution, as it points more towards trend cues rather than deterministic conclusions. Secondly, despite our efforts to control dietary regions in patient recruitment and perioperative management BMI、 Heterogeneity of variables such as surgical procedures and antibiotic use, such as all patients coming from the same region and having similar dietary habits; During the perioperative period, the same treatment group from the same center is responsible for ensuring that the differences between surgery and medication are basically eliminated. But these measures can only partially alleviate, and cannot completely eliminate the impact of confounding factors. We did not directly record individualized dietary intake details, antibiotic exposure history, or lifestyle habits related to gut microbiota regulation, and these unmeasured factors may still leave significant interference traces in microbial composition. In addition, even if medication is standardized during the perioperative period, implicit variables such as individual differences in drug metabolism responses may also affect the microbiota, and we lack specific quantitative data on these processes. Therefore, the association between observed changes in microbiota and thyroid function cannot be ruled out as distorted by residual confounding. Finally, this study did not establish a parallel healthy control group. Our reliance is on asynchronous comparisons with external data from previously published literature, and there are differences in sample processing, sequencing platforms, and data analysis processes between different studies. Batch effects and time drift may introduce systematic biases. Although we assume baseline differences in microbiota between cancer patients and healthy individuals based on sufficient evidence from previous studies, this indirect comparison cannot effectively control for external factors such as time, experimental conditions, and population characteristics. Therefore, when attributing specific changes in microbiota caused by surgery, the level of evidence chain is significantly limited.

Based on the preliminary findings of this study, further work will be carried out from the following aspects to more rigorously verify the role of gut microbiota in postoperative functional regulation of thyroid cancer: firstly, expand the prospective cohort (≥ 100 cases), increase preoperative, early postoperative, and long-term follow-up time points, and include healthy control and benign thyroid surgery control groups to distinguish between anatomical trauma, disease status, and tumor specific microbial changes. Secondly, strengthen diet BMI、 Standardized recording of confounding factors such as antibiotics and unified control of medication to minimize the interference of external variables such as diet and medication on microbial composition.. Thirdly, shotgun metagenomic sequencing was used instead of 16S amplicons to obtain strain and functional gene information. Combined with serum metabolomics (bile acids, short chain fatty acids, etc.), a correlation network between microbial metabolites and thyroid hormones was constructed. Fourthly, targeting key differentially expressed bacterial genera (such as *Bacteroidetes*), an anaerobic co culture system was used to verify their effects on the uptake of iodine and selenium by thyroid follicular cells. Through antibiotic clearance or fecal microbiota transplantation mouse models, combined with post thyroidectomy intervention, molecular pathways such as deiodinase activity and enterohepatic circulation were directly analyzed. Through the multidimensional and multi-level promotion mentioned above, we hope to make up for the shortcomings of current research in sample size, control, confounding control, and mechanism depth.In summary, this exploratory study has certain limitations. Future studies can address these limitations by increasing the sample size, adding a third time point and using other methods. Currently, research focusing on this particular angle remains scarce. Therefore, this study’s findings are expected to provide valuable references and inspiration for subsequent large-scale studies and advancements in clinical applications.

## Conclusions

In this exploratory paired observational study, patients with papillary thyroid carcinoma showed short-term postoperative changes in gut microbiota composition, diversity, and predicted functional pathways. Several bacterial taxa were associated with thyroid hormone-related biomarkers. These findings support a potential relationship between the perioperative gut microbiome and thyroid hormone regulation, but larger longitudinal studies with appropriate controls are needed to validate these associations and clarify causality.

## Supporting information

S1 Fig Flow chart of the experiment. (TIF)

S2 Fig Gut microbiota differences based on metagenomic sequencing at the species level: (A) β-diversity based on the Bray–Curtis distance; (B) PCoA scatter plot; (C) ANOSIM analysis; (D, E) Pie chart of community analysis at the species level for the BF group (D) and FF group (E). FF stands for pre-operative group, and BF stands for post-operative group. ANOSIM: Analysis of Similarities.
(TIF)

S1 Table Patient baseline characteristics. (DOCX)

S2 Table The correlation coefficient and adjusted *p*-value in Fig 3C. (XLSX)

S3 Table The LDA-scores and *p*-value in Fig 4A. (CSV)

S4 Table The pathways of significant enrichment, ReporterScore, *p*-value and enrichment directions in Fig 4B. (CSV)
