# Supplementary material for: Metagenomic analysis of gut microbiota and its correlation with thyroid hormones in papillary thyroid cancer before and after operation
Source: PLoS One. 2026 Aug 24;21(8):e0356770. doi: 10.1371/journal.pone.0356770 (PMC13502680; doi:10.1371/journal.pone.0356770)
Supplement: Supplementary file 1 [file pone.0356770.s003.docx]

**TABLE S1** Patient baseline characteristics

| Sample | Gender | Age | BMI | Surgical approach | Postoperative thyroid hormone medication |
| --- | --- | --- | --- | --- | --- |
| 1 | Male | 27 | 18.339 | Total thyroidectomy of right lobe and isthmus | Levothyroxine Sodium Tablets（Euthyrox） 75ug qd |
| 2 | Male | 35 | 21.63 | Total thyroidectomy of right lobe and isthmus | Levothyroxine Sodium Tablets（Euthyrox） 75ug qd |
| 3 | Male | 47 | 22.888 | Total thyroidectomy of left lobe and isthmus | Levothyroxine Sodium Tablets（Euthyrox） 75ug qd |
| 4 | Male | 25 | 22.783 | Total thyroidectomy of left lobe and isthmus | Levothyroxine Sodium Tablets（Euthyrox） 75ug qd |
| 5 | Male | 54 | 21.887 | Total thyroidectomy of right lobe and isthmus | Levothyroxine Sodium Tablets（Euthyrox） 75ug qd |
| 6 | Male | 42 | 21.008 | Total thyroidectomy of left lobe and isthmus | Levothyroxine Sodium Tablets（Euthyrox） 75ug qd |
| 7 | Male | 33 | 21.929 | Total thyroidectomy of right lobe and isthmus | Levothyroxine Sodium Tablets（Euthyrox） 75ug qd |
| 8 | Male | 52 | 19.596 | Total thyroidectomy of left lobe and isthmus | Levothyroxine Sodium Tablets（Euthyrox） 75ug qd |
| 9 | Male | 47 | 22.663 | Total thyroidectomy of left lobe and isthmus | Levothyroxine Sodium Tablets（Euthyrox） 75ug qd |
| 10 | Male | 35 | 21.358 | Total thyroidectomy of left lobe and isthmus | Levothyroxine Sodium Tablets（Euthyrox） 75ug qd |
| 11 | Female | 42 | 20.546 | Total thyroidectomy of right lobe and isthmus | Levothyroxine Sodium Tablets（Euthyrox） 75ug qd |
| 12 | Female | 35 | 21.338 | Total thyroidectomy of right lobe and isthmus | Levothyroxine Sodium Tablets（Euthyrox） 75ug qd |
| 13 | Female | 44 | 21.719 | Total thyroidectomy of right lobe and isthmus | Levothyroxine Sodium Tablets（Euthyrox） 75ug qd |
| 14 | Female | 58 | 24.035 | Total thyroidectomy of left lobe and isthmus | Levothyroxine Sodium Tablets（Euthyrox） 75ug qd |
| 15 | Female | 61 | 18.827 | Total thyroidectomy of right lobe and isthmus | Levothyroxine Sodium Tablets（Euthyrox） 75ug qd |
| 16 | Female | 36 | 19.195 | Total thyroidectomy of right lobe and isthmus | Levothyroxine Sodium Tablets（Euthyrox） 75ug qd |
| 17 | Female | 38 | 21.795 | Total thyroidectomy of left lobe and isthmus | Levothyroxine Sodium Tablets（Euthyrox） 75ug qd |
| 18 | Female | 58 | 21.977 | Total thyroidectomy of left lobe and isthmus | Levothyroxine Sodium Tablets（Euthyrox） 75ug qd |
| 19 | Female | 29 | 20.812 | Total thyroidectomy of left lobe and isthmus | Levothyroxine Sodium Tablets（Euthyrox） 75ug qd |
| 20 | Female | 59 | 19.228 | Total thyroidectomy of left lobe and isthmus | Levothyroxine Sodium Tablets（Euthyrox） 75ug qd |

All patients had early-stage low-risk tumors and did not take antibiotics before surgery.
